# Polysulfone/Graphene Oxide Mixed Matrix Membranes for Improved CO_2_/CH_4_ Separation

**DOI:** 10.3390/membranes15120386

**Published:** 2025-12-18

**Authors:** Mustafa Alsaady, Sharjeel Waqas, Mohammed A. Almarshoud, Khuram Maqsood, Aymn Abdulrahman, Yuying Yan

**Affiliations:** 1 Chemical Engineering Department, University of Jeddah, Jeddah 23890, Saudi Arabia; 2 Interdisciplinary Research Center for Membranes and Water Security, King Fahd University of Petroleum and Minerals, Dhahran 31261, Saudi Arabia; 3 Civil and Environmental Engineering Department, University of Jeddah, Jeddah 23890, Saudi Arabia; 4 Fluids and Thermal Engineering Research Groups, Faculty of Engineering, University of Nottingham, Nottingham NG7 2RD, UK

**Keywords:** CO_2_/CH_4_ separation, mixed matrix membranes, GO filler, CO_2_ plasticization, Polysulfone

## Abstract

This research focuses on developing and optimizing mixed matrix membranes (MMMs) by incorporating graphene oxide (GO) into a polysulfone (PSF) matrix to enhance the separation performance of CO_2_ and CH_4_. The morphology and gas separation performance of the MMMs were systematically characterized. The incorporation of GO enhanced gas permeation and CO_2_/CH_4_ selectivity, as evaluated using a gas permeation setup. Notably, the PSF/GO-0.3 wt.% membrane exhibited superior performance, achieving a CO_2_ permeability of 21.63 Barrer, among the highest reported for PSF-based MMMs. Additionally, the membrane demonstrated a CO_2_/CH_4_ selectivity of 14.32, highlighting its effectiveness in distinguishing between the two gases, which is essential for carbon capture and natural gas decontamination applications. The uniform distribution of GO within the polymer matrix contributed to the membrane’s enhanced performance. Furthermore, the MMMs exhibited outstanding resistance to CO_2_ plasticization, with the PSF/GO-0.3 wt.% membrane maintaining its performance at pressures up to 10 bar, a significant improvement over the pristine PSF membrane, which failed at 4 bar. The improved plasticization resistance is ascribed to the reinforcing effect of GO, which stabilizes the polymer matrix, minimizing CO_2_-induced swelling. The PSF/GO-0.3 wt.% membrane exhibited exceptional CO_2_ permeability, selectivity, and plasticization resistance, making it a viable alternative for industrial gas separation applications and outperforming previously reported PSF-based MMMs.

## 1. Introduction

Anthropogenic carbon dioxide (CO_2_) emissions have become a critical global issue, driving climate change and environmental degradation [1]. Efforts to mitigate global greenhouse gas emissions have increasingly focused on developing advanced gas separation technologies, particularly those aimed at capturing CO_2_ from industrial gas streams such as those encountered in natural gas processing, syngas purification, and carbon capture and storage (CCS) systems [2]. Among the available separation techniques, including cryogenic distillation, adsorption, absorption and membrane-based gas separation has gained significant attention due to its advantages of low energy consumption, modularity, ease of scale-up, and minimal environmental footprint [3,4]. Membrane processes eliminate the need for regenerable chemical solvents, thereby reducing both operational costs and secondary waste generation, making them particularly attractive for large-scale CO_2_ capture and natural gas sweetening applications [5,6].

Mixed matrix membranes (MMMs) have materialized as a promising next-generation solution. MMMs combine inorganic fillers, such as metal organic frameworks (MOFs), zeolites, covalent organic frameworks (COFs), and graphene-based nanomaterials, with polymeric matrices to synergistically exploit the permeability of the polymer and the selectivity of the filler [7,8,9]. This hybrid configuration provides new opportunities to surpass the classical Robeson trade-off between permeability and selectivity and to tailor molecular transport pathways through optimized polymer filler interfaces [10,11]. Previous researchers have investigated the incorporation of functionalized inorganic materials into polymeric matrices to overcome this limitation, producing MMMs with improved gas separation [12,13].

Graphene oxide (GO), a monolayer form of graphite oxide, is obtained from the graphite’s extensive oxidation process. GO is typically obtained by exfoliating graphite oxide, producing thin, wrinkled two-dimensional carbon sheets [14]. Its structure is characterized by hydroxyl and carboxyl functional groups distributed across its edges and basal planes. These functional groups enable GO sheets to exhibit high CO_2_ sorption capacity. This property makes fabricating membranes from GO suspensions more straightforward and scalable than other filler materials [15].

Polysulfone (PSF) was selected as the polymer matrix in this work, owing to its superior stability and longevity compared to highly permeable polymers like polymers of intrinsic microporosity, which suffer from significant physical ageing [16,17]. PSF resistance to ageing ensures that membranes from it maintain sustainable performance and a long lifespan, which is crucial for industrial applications where consistent efficiency is essential [18]. Moreover, PSF is a commercially available, cost-effective polymer with excellent physicochemical properties, high thermal stability, and resistance to chemicals like bases, acids, and chlorine. These characteristics make PSF versatile and suitable for diverse operating conditions. Additionally, its good mechanical strength and processability enable the production of robust, durable membranes with enhanced gas separation, making it a valuable material in membrane separation technology [19].

Several researchers have reported the PSF/GO MMMs for CO_2_/CH_4_ separation [20,21,22]. These membranes exhibited enhanced CO_2_/CH_4_ selectivity compared to pure polymer membranes, owing to synergistic interactions within the mixed matrix structure [23]. Wong et al. [24] introduced an interfacial polymerization method to incorporate thin-film nanocomposite (TFN) membranes with GO layers. The membranes exhibited exceptional separation performance at a GO filler concentration of 0.5 g/L, achieving high selectivity with separation factors of 41 for CO_2_/N_2_ and 25 for CO_2_/CH_4_. Despite the impressive selectivity, the membrane showed a relatively moderate CO_2_ permeance of 92.4 gas permeation units (GPU). Berean et al. [25] developed a series of polydimethylsiloxane (PDMS)/graphene composite membranes, incorporating multilayer graphene nanoplatelets as the dispersed phase within a continuous PDMS matrix. Remarkably, the introduction of graphene flakes significantly enhanced the gas permeation properties of the membranes (for gases such as N_2_, CO_2_, Ar, and CH_4_), in contrast to those composed solely of PDMS. The improvement in gas transport was attributed to the formation of interfacial voids between the polymer chains and graphene flakes, which increased free volume within the composite membranes and thereby facilitated enhanced molecular diffusion. Despite notable advancements in MMMs, the large scale application remains constrained by several critical challenges. These include maintaining long term operational stability, achieving consistent large-scale production, and reducing overall fabrication costs [26,27]. Overcoming these barriers requires a more comprehensive understanding of the relationships between membrane structure and performance, along with careful control of fabrication parameters to promote uniform filler distribution and strong polymer/filler interfacial bonding.

The novelty of this study lies in the fabrication of PSF/GO MMMs for CO_2_/CH_4_ separation, a combination that has been minimally explored in the literature. By incorporating GO into PSF membranes, this research aims to enhance gas separation, with a particular focus on improving selectivity and permeability for CO_2_/CH_4_ separations, which remains a critical challenge in membrane technology. This study offers new insights into the potential of PSF/GO MMMs for advanced gas separation applications. Accordingly, this study aims to optimize the composition and morphology of PSF/GO MMMs and to comprehensively characterize their structural, morphological, and surface properties. Additionally, the research aims to systematically evaluate the gas transport performance of the developed membranes, with a particular emphasis on CO_2_ permeability and CO_2_/CH_4_ selectivity. Through these investigations, the study seeks to enhance the understanding and design of high performance MMMs for CO_2_/CH_4_ separation, with potential implications for natural gas purification and post-combustion carbon capture technologies.

## 2. Materials and Methods

### 2.1. Materials

PSF (weight-average molecular weight (Mw) ~ 35 kDa; and number-average molecular weight (Mn) ~ 16 kDa), along with graphite powder, were procured from Sigma-Aldrich, St. Louis, MO, USA. Sulfuric acid (H_2_SO_4_, molecular weight = 98.079 g/mol, purity = 95–98%), hydrogen peroxide (H_2_O_2_, molecular weight = 34.01 g/mol, purity = 35%), and potassium permanganate (KMnO_4_, molecular weight = 158.034 g/mol) were acquired from Merck, Darmstadt, Germany. N, N-Dimethylformamide (DMF, purity > 99%), nitric acid (HNO_3_, concentration = 65–70%), and sodium nitrate (NaNO_3_, molecular weight = 84.99 g/mol) were sourced from Sigma-Aldrich, St. Louis, MO, USA. Silver nitrate (AgNO_3_) was obtained from Scharlab, Barcelona, Spain. All the chemicals were analytically graded and were used as received.

### 2.2. Methods

#### 2.2.1. Synthesis of Graphene Oxide

GO was synthesized using a modified Hummers method [28]. A mixture containing 10 g of graphite powder and 3 g of NaNO_3_ was added to 100 mL of concentrated H_2_SO_4_, and the mixture was stirred while maintaining the temperature at 5 °C. Subsequently, 12 g of KMnO_4_ was added gradually at 20 °C, and the reaction mixture was stirred for 5 min before increasing the temperature to 35 °C for 30 min. To control the reaction temperature, 500 mL of distilled water was gradually introduced, after which an additional 25 mL of water and 10 mL of H_2_O_2_ were added at 60 °C to ensure the complete removal of any remaining KMnO_4_. The mixture was then centrifuged at 6500 rpm for 45 min, and the solid obtained was repeatedly washed with a copious amount of 5% hydrochloric acid and DI water to remove impurities. The final GO product, a dark brown powder, was obtained after freeze drying and stored for further use.

#### 2.2.2. Mixed Matrix Membrane Fabrication

MMMs with different GO loadings were prepared via solution blending. PSF was first dissolved in DMF under continuous stirring for 18 h to obtain a homogeneous polymer solution. The resulting solution was degassed at 25 °C overnight to remove entrapped air bubbles. Separately, GO particles (0.1–0.5 wt.%) were dispersed in DMF through alternating cycles of magnetic stirring and ultrasonication to achieve uniform dispersion. Approximately 10 wt.% of the PSF/DMF solution was then added to the GO/DMF dispersion to pre-wet and prime the GO particles, followed by 30 min of stirring and sonication. The remaining PSF/DMF solution was then added to the primed dispersion and stirred and sonicated for an additional 30 min to ensure complete homogenization. The final casting solution was stirred vigorously for 1 h and then cast onto a leveled Petri dish. After solvent evaporation, the membranes were carefully peeled off and dried at ambient temperature. The average membrane thickness, measured using a digital micrometer, was 0.28 ± 0.03 mm. The fabricated PSF/GO MMMs were then characterized and evaluated for gas permeation performance.

### 2.3. Membrane Characterization

#### 2.3.1. Scanning Electron Microscope (SEM) and Energy Dispersive X-Ray Spectroscopy (EDX)

The surface and cross-sectional morphology of the fabricated MMMs were examined using a scanning electron microscope (SEM), Tescan Clara, Czech Republic. Samples were first immersed in liquid nitrogen and fractured to obtain clean cross-sections with uniform morphology. Prior to imaging, each sample was sputter-coated with a thin gold layer to improve image resolution and surface conductivity. The elemental composition and distribution of the prepared MMMs were analyzed using energy dispersive X-ray spectroscopy (EDX), Tescan Clara, Czech Republic. EDX spectra were collected from micrographs corresponding to a field width of approximately 10 µm.

#### 2.3.2. Fourier Transform Infrared Spectroscopy (FTIR)

Fourier transform infrared (FTIR) spectroscopy (PerkinElmer Spectrum One, Shelton, CT, USA) was utilized to detect the functional groups in the membrane samples using the KBr pellet technique. A small amount (typically 1–2 mg) of the solid sample with approximately 10 mg of dry, spectral grade KBr powder. This mixture is homogenized to ensure an even sample distribution within the KBr matrix. The ground mixture is then placed in a die to form pellets. Transparent and crack-free KBr pellets were prepared to ensure accurate spectral measurements. The analysis was conducted in transmittance mode over a wavenumber range of 4000–500 cm^−1^. The collected interferograms were processed using the Fourier transform algorithm to obtain the corresponding spectra, and baseline corrections were applied to eliminate artefacts. The absorption peaks were interpreted to identify characteristic molecular vibrations and chemical bonds, and a quantitative assessment was performed by analyzing peak intensities or areas applicable.

#### 2.3.3. Thermogravimetric Analysis (TGA)

Thermogravimetric analysis (TGA) of the pristine polymers and the fabricated MMMs was conducted using a Mettler Toledo TGA/SDTA 851e instrument, Columbus, OH, USA. Samples were heated at 10 °C min^−1^ from ambient temperature to 800 °C under a nitrogen flow of 15 mL min^−1^ to maintain an inert atmosphere.

#### 2.3.4. Differential Scanning Calorimetry (DSC)

Differential scanning calorimetry (DSC, Q2000) was employed to assess the glass transition temperature (Tg) of the prepared samples. Approximately 3 mg of each specimen was sealed in an aluminum pan and subjected to thermal analysis. An initial heating cycle to 350 °C at 20 °C min^−1^ was applied to remove residual moisture and erase thermal history, followed by quenching to 30 °C and reheating under identical conditions. The Tg was determined from the heat flow temperature curves using the onset method.

### 2.4. Gas Permeation Testing

The constant volume variable pressure technique evaluated the single gas permeation performance. A circular membrane sample was prepared and installed into a membrane cell, providing an effective permeation area of 1 cm^2^. The assembled module was subsequently integrated into a gas permeation test apparatus. Before initiating single gas measurements, the system was evacuated using a vacuum pump via the upstream purge stream to eliminate residual adsorbed gases and moisture. Subsequently, the system was flushed with the test gas to displace any remaining traces of humid air. The permeation experiments were conducted across a pressure range of 2 to 12 bars. Pure gas was introduced in the sequence of CH_4_, followed by CO_2_. The test apparatus was evacuated to remove residual gas molecules and purged with the respective test gas for each transition between feed gases. To ensure reproducibility, each measurement was performed in triplicate, and the reported values represent the average of the three replicates. The CO_2_ and CH_4_ permeability and CO_2_/CH_4_ selectivity of the membrane were obtained using Equations (1) and (2), respectively [29].

(1) $$\frac{P_{x}}{L}=\frac{Q}{A∆P}$$

where Px, L, A, ΔP, and Q represent the permeability of x gas (x = CO_2_ or CH_4_), membrane thickness, area, pressure drop, and permeate flow rate, respectively. PCO_2_ and PCH_4_ refer to the permeability of the sample membrane to CO_2_ and CH_4_, measured in Barrer.

(2) $$\alpha_{{CO}_{2}/{CH}_{4}}=\frac{P_{{CO}_{2}}}{P_{{CH}_{4}}}$$

## 3. Results and Discussion

### 3.1. SEM Analysis

**Figure 1:**
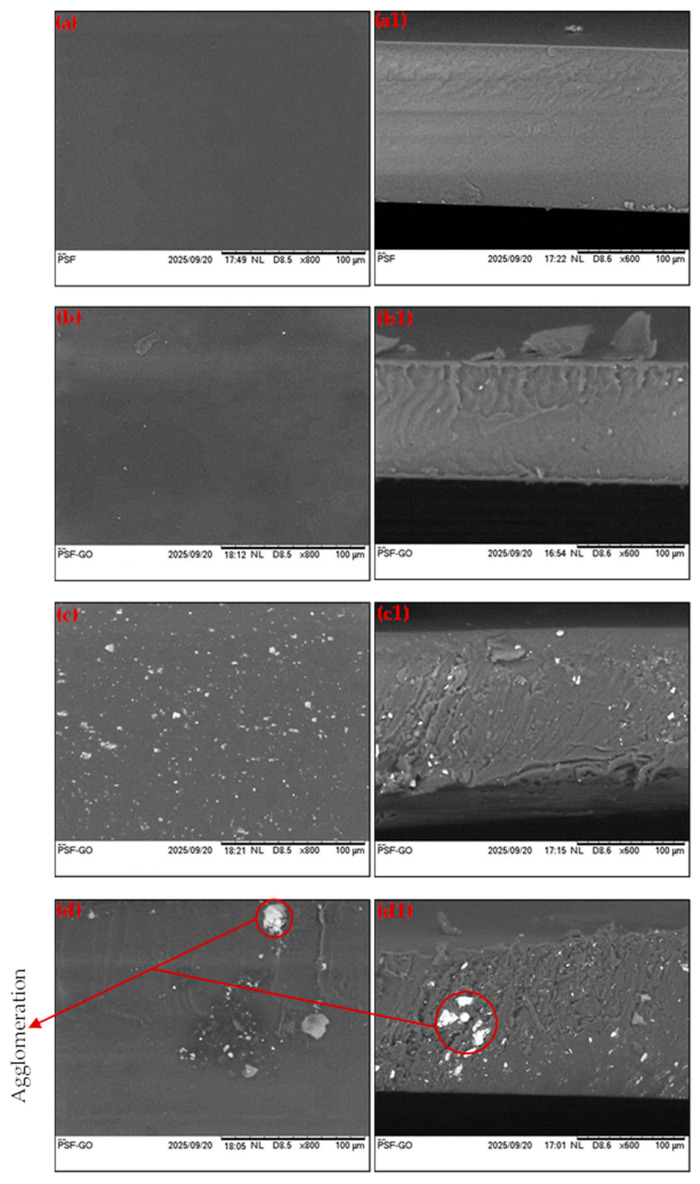
SEM images (surface and cross-section) of resultant membranes (**a**,**a1**) 0 wt.%, (**b**,**b1**) 0.1 wt.%, (**c**,**c1**) 0.3 wt.%, and (**d**,**d1**) 0.5 wt.%.

The surface and cross-sectional morphologies of the prepared membranes are presented in Figure 1. Figure 1(a,a1) show the top-view and cross-sectional SEM micrographs of the pristine PSF membrane, respectively, and Figure 1(b–d,b1–d1) corresponds to PSF/GO MMMs containing different GO loadings. As the GO content increases from 0.1 wt.% to 0.5 wt.%, a noticeable increase in the number of dispersed particles on the membrane surface is observed, indicating successful incorporation of the filler into the polymer matrix.

The pristine PSF membrane (Figure 1(a,a1)) exhibits a smooth and homogeneous surface with a dense, defect-free cross-section, indicating uniform polymer film formation during casting. This morphology reflects the typical characteristics of glassy PSF membranes prepared by solvent evaporation. Upon incorporation of GO, distinct morphological variations are observed. At 0.1 wt.% GO loading as shown in Figure 1(b,b1), the membrane surface remains relatively smooth, with a few bright domains attributed to well-dispersed GO nanosheets embedded within the PSF matrix. The cross-sectional image reveals the formation of shallow, finger-like voids, likely due to the enhanced phase inversion kinetics enabled by GO’s hydrophilic nature.

At an intermediate GO content of 0.3 wt.% (Figure 1(c,c1)), the surface becomes slightly rougher, with a noticeable increase in the number of dispersed particles, suggesting a more homogeneous dispersal of filler through the polymer matrix. This uniformity indicates strong interfacial compatibility between GO and PSF, which can be attributed to hydrogen bonding between the hydroxyl and oxygen containing groups on GO and the sulfone or ether linkages of the PSF chains. Such interactions enhance filler dispersion and promote better interfacial adhesion, thereby minimizing the formation of non-selective voids or interfacial defects. The resulting morphology is compact and well-integrated, demonstrating that moderate GO loading promotes a synergistic interaction between the polymer and filler.

At higher GO content (0.5 wt.%; Figure 1(d,d1)), agglomeration of GO nanosheets becomes apparent on both the membrane surface and cross-section. These agglomerates form due to the strong van der Waals attraction between GO sheets, which increases with higher concentrations. Agglomeration leads to the generation of localized defects and interfacial voids, which may act as non-selective transport pathways, thereby reducing gas separation efficiency despite a possible increase in permeability.

Overall, the SEM observations confirm the effective integration of GO into the PSF, with the degree of dispersion highly dependent on filler concentration. The presence of functional hydroxyl groups on GO enhances polymer/filler compatibility via hydrogen bonding, thereby promoting the formation of uniform, defect-free membrane structures. The reduction of non-selective voids within the MMMs consequently enhances gas pair selectivity, allowing the membrane to facilitate CO_2_ transport while preferentially rejecting CH_4_. Hence, the combined effects of improved interfacial compatibility and GO’s selective affinity for CO_2_ contribute to superior gas separation performance.

### 3.2. Elemental and EDX Analysis

**Figure 2:**
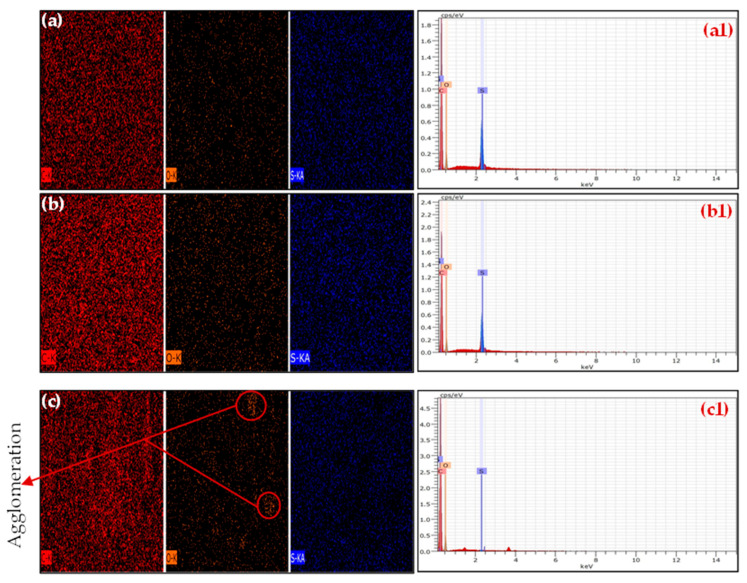
EDX surface mapping and spectra of resultant MMMs (**a**,**a1**) GO-0.1 wt.%, (**b**,**b1**) GO-0.3 wt.%, and (**c**,**c1**) GO-0.5 wt.%.

The elemental composition and distribution of the PSF/GO MMMs were examined using EDX, as shown in Figure 2. The mapping images and corresponding spectra reveal that carbon (C), oxygen (O), and sulfur (S) are the principal elements in all samples. The uniform dispersion of oxygen signals across the membrane surface indicates a homogeneous distribution of GO nanosheets within the PSF matrix, particularly at moderate loadings (0.3 wt.%, Figure 2(b,b1)). This uniformity supports the SEM observations and suggests strong interfacial interaction between the hydrophilic GO sheets and the PSF. The oxygen rich regions correspond to sites where hydrogen bonding likely occurs between the GO functional groups and the sulfone or ether linkages of PSF, thereby promoting strong polymer/filler adhesion and reducing the likelihood of interfacial voids. At higher filler loading (0.5 wt.%), as shown in Figure 2(c,c1), a slight clustering of oxygen signal intensity was observed in localized regions (as also seen in the SEM images), indicating partial aggregation of GO nanosheets at excessive concentrations. Such agglomeration may lead to uneven element distribution and potential defects in the membrane structure.

Overall, the EDX spectra validate the elemental composition of the PSF/GO MMMs and confirm the incorporation and dispersion of GO within the PSF matrix. The consistent presence of carbon (C), oxygen (O), and sulfur (S), along with the increasing oxygen intensity with increasing GO content, provides strong evidence of GO integration. These findings further support the morphological analysis, indicating that uniform GO dispersion enhances compatibility and structural integrity, key factors that influence gas separation performance.

### 3.3. FTIR Analysis

**Figure 3:**
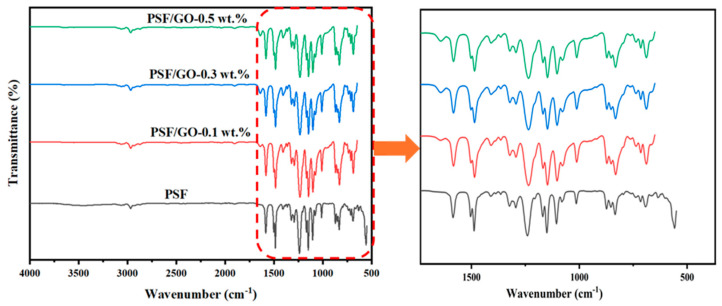
FTIR of the prepared PSF and PSF/GO MMMs.

The FTIR spectra of pristine PSF and PSF/GO MMMs with varying GO loadings (0.1, 0.3, and 0.5 wt.%) are presented in Figure 3. The characteristic absorption bands of PSF and the variations introduced by GO incorporation are clearly observed across the spectra. The broad absorption band observed between 3000 and 3200 cm^−1^ is attributable to the O–H stretching vibrations arising from surface hydroxyl functionalities or physisorbed water on the GO layers. The presence of multiple overlapping O–H stretching modes indicates that hydroxyl groups on the GO surface likely exist in diverse chemical environments, including tertiary alcohol, phenolic, and carboxylic configurations [28,30]. For the pristine PSF membrane, the major peaks appearing at approximately 1585 cm^−1^ are attributed to the aromatic C=C stretching vibration within the benzene rings of the PSF backbone. The bands located around 1488 and 1401 cm^−1^ correspond to C–C stretching in the aromatic structure. The peak observed at 1322 cm^−1^ is associated with the asymmetric stretching vibration of the sulfone (O=S=O) group, while the strong bands in the range of 1240–1150 cm^−1^ are assigned to the asymmetric stretching of the ether (C–O–C) linkages. Furthermore, the peaks between 1100 and 950 cm^−1^ are ascribed to symmetric O=S=O stretching and aromatic C–H in plane bending vibrations, whereas the absorption bands in the region of 830–700 cm^−1^ are due to aromatic C–H out of plane bending. These characteristic peaks confirm the typical structural features of PSF, including aromatic rings, ether linkages, and sulfone functionalities.

The enlarged portion of the spectra (highlighted in red in Figure 3) focuses on the region between 1500 and 500 cm^−1^, where subtle but significant changes can be detected following the incorporation of GO into the PSF matrix. With increasing GO concentration, noticeable shifts and slight broadening of the peaks at around 1240–1150 cm^−1^ and 1100 cm^−1^ are observed. These changes can be attributed to intermolecular interactions between the PSF chains and the oxygen containing functional groups (e.g., hydroxyl, carbonyl, and carboxyl) on the GO surface. These modifications are likely due to the development of intermolecular interactions between the PSF polymer chains and the oxygenated functional groups on the GO surface, including hydroxyl, carbonyl, and carboxyl groups. Additionally, a gradual increase in intensity around 1030–1070 cm^−1^ is evident, which corresponds to the C–O stretching vibrations of the hydroxyl and epoxy groups on GO. This observation indicates that GO has been successfully incorporated and uniformly dispersed within the PSF matrix, thereby enhancing interfacial compatibility.

Moreover, minor variations in the spectral region of 830–700 cm^−1^ suggest possible π–π stacking interactions between the aromatic rings of PSF and the sp^2^-hybridized carbon network of GO nanosheets. These interactions indicate that GO forms non-covalent bonds with the PSF matrix, enhancing interfacial adhesion and reducing the likelihood of interfacial voids within the MMM structure. The presence of these interactions also indicates potential hydrogen bonding and dipole–dipole interactions between the polar groups of PSF (sulfone and ether) and the oxygen functionalities of GO. Overall, the FTIR spectra confirm that the incorporation of GO does not disrupt the fundamental structure of PSF but rather introduces additional interaction sites, promoting strong interfacial bonding between the polymer chains and GO nanosheets. These interfacial interactions are expected to improve the physicochemical performance of the resultant MMMs by enhancing structural integrity and potentially the hydrophilic character of the membrane surface.

### 3.4. TGA

**Figure 4:**
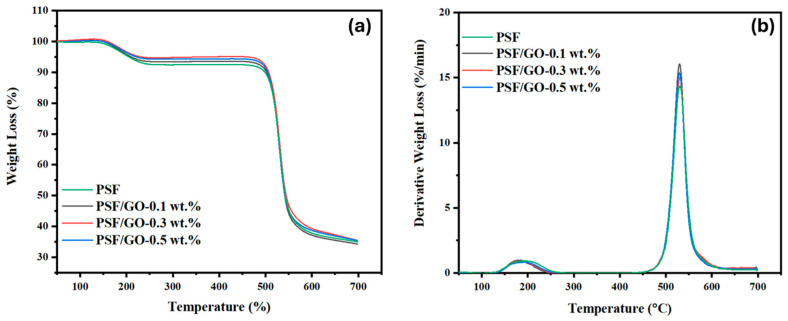
Thermal gravimetric analysis of prepared PSF and PSF/GO membrane with (**a**) weight loss, (**b**) derivative of weight loss summary.

The thermal stability of the prepared membranes is shown in Figure 4. The initial degradation stage, occurring between 150 and 220 °C, corresponds to minor chain scission events. The second stage, observed between 230 and 500 °C, is associated with irregular chain terminations. The third stage, above 550 °C, involves random cleavage of C-C bonds within the repeating structural units. The pristine polymer membrane exhibited a decomposition temperature of approximately 500 °C, consistent with previously reported values. All MMMs containing GO showed a slight initial weight loss, followed by a major degradation stage between 520 and 560 °C. The pronounced mass loss between 500 °C and 700 °C is attributable to the thermal decomposition of the remaining, more thermally stable oxygen containing functional groups within the GO framework, accompanied by the pyrolytic degradation of the destabilized carbon skeleton. The derivative thermogravimetric (DTG) curves, presented in Figure 4b, provide further confirmation of the thermal behaviour. Two distinct peaks were observed for all MMMs: the first transition (Tp_1_), appearing between 150 and 230 °C, is attributed to the evaporation of residual water or solvents, while the second transition (Tp_2_), occurring near 520 °C, corresponds to the degradation of the polymer backbone together with the breakdown of the GO crystalline framework.

**Figure 5:**
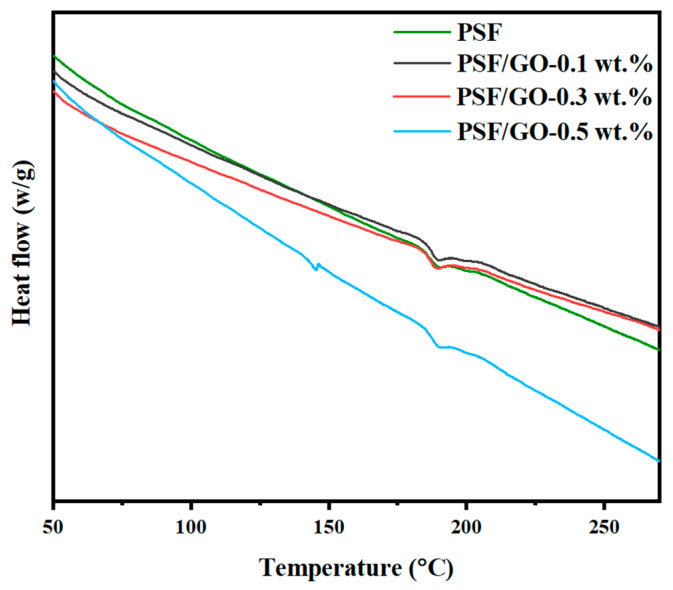
DSC analysis of resultant MMMs.

GO exhibits distinctive thermal characteristics arising from its oxygen containing functional groups, such as hydroxyl, epoxy, and carbonyl moieties, which decompose at intermediate temperatures while its graphitic backbone remains structurally robust at higher temperatures. When incorporated into PSF, GO influences the overall thermal behavior of the composite by restricting polymer chain mobility and delaying the onset of thermal degradation. This effect is often manifested as an elevation in the glass transition temperature (Tg) (Figure 5) and a shift in decomposition profiles to higher temperatures. The underlying mechanism is typically attributed to strong interfacial interactions between the aromatic domains of PSF and the π-conjugated surface of GO, in addition to hydrogen bonding between polymer functional groups and oxygenated sites on GO. The incorporation of GO into the polymer matrix resulted in a gradual increase in Tg, suggesting a relatively uniform dispersion of the nanoparticles. This enhancement is attributed to strengthened interfacial interactions between the polymer chains and the incorporated nanoparticles, such as ester groups, methyl groups, and carbonyl (C=O) groups, which restrict chain mobility and elevate the thermal rigidity of the membranes.

Overall, incorporating GO led to a measurable improvement in the membranes’ thermal resistance, as indicated by the upward shift in decomposition temperatures relative to pristine PSF. A gradual increase in GO loading was accompanied by a further elevation in degradation onset, underscoring its role in retarding polymer chain breakdown. While CO_2_/CH_4_ separation in natural gas processing is typically performed under moderate thermal conditions, the availability of membranes with improved thermal endurance offers clear advantages in terms of structural integrity, long-term durability, and operational safety. Moreover, such improvements expand the potential applicability of these membranes to more demanding separation processes involving elevated temperatures or aggressive environments.

### 3.5. DSC Analysis

DSC was used to evaluate the thermal characteristics of the fabricated membrane samples. As shown in Figure 5, all membranes exhibited a single glass transition temperature (Tg) of 186–190 °C. The pristine PSF membrane displayed a Tg of 186.83 °C, whereas the Tg values of PSF/GO-0.1 wt.%, PSF/GO-0.3 wt.%, and PSF/GO-0.5 wt.% were 188.45 °C, 189.75 °C, and 190.3 °C, respectively. The progressive shift in Tg with increasing GO incorporation indicates that GO incorporation into the polymer matrix restricts chain mobility, thereby enhancing the thermal rigidity of the membranes. The observed increase in Tg can be ascribed to strong interfacial interactions between PSF and GO. Similar findings have been reported in earlier studies. For instance, Zahri et al. [31] incorporated 0.25 wt.% GO into a PSF matrix to fabricate mixed matrix membranes for gas permeation, and the elevated Tg values were linked to enhanced interactions between the polymer chains and GO. This effect was due to the abundance of oxygenated functional groups on the planar surface of GO, which form bridging bonds on both sides of the sheet, thereby restricting chain mobility and increasing the glass transition temperature (Tg). In a separate study, Wan et al. [32] investigated the interfacial reinforcement of GO in polymer-based nanocomposites, including PLLA, PCL, PS, and PE. They reported that the Tg of pristine polystyrene (PS) (73.5 °C) increased to 86.4 °C with 1 wt.% GO, as strong interfacial interactions limit the mobility of PS chains adsorbed on the GO surface. This restriction was attributed to π–π stacking interactions between the polymer’s aromatic rings and the graphite domains of GO. Given the structural similarity between PSF and PS, it is reasonable to infer that the aromatic rings in PSF undergo comparable interactions with GO, resulting in analogous thermal reinforcement. Furthermore, higher GO loadings are associated with more pronounced increases in Tg, confirming GO’s reinforcing effect on the polymer structure.

### 3.6. Gas Separation Performance

**Figure 6:**
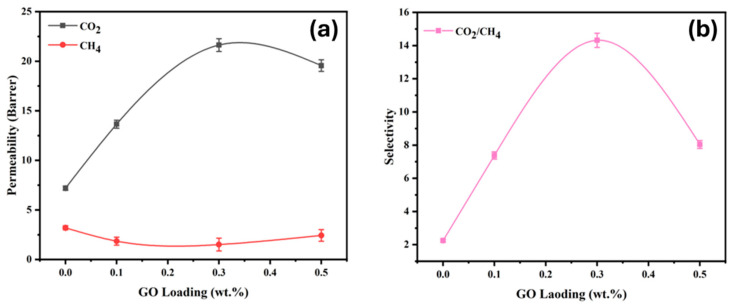
Effect of GO filler ratio on gas (**a**) permeability and (**b**) selectivity.

The gas separation performance of PSF and PSF/GO membranes with varying GO concentrations (0.1, 0.3, and 0.5 wt.%) is illustrated in Figure 6. The permeability of CO_2_ and CH_4_, as well as the corresponding CO_2_/CH_4_ ideal selectivity, were investigated to evaluate the influence of GO incorporation on the gas transport behaviour of the membranes. As shown in Figure 6a, the CO_2_ permeability increases progressively with loading up to 0.3 wt.%, reaching a maximum value of approximately 21.63 Barrer, which is more than twice that of the pristine PSF membrane (7.20 Barrer). It is important to note that pristine PSF membranes do not exhibit a universal selectivity value; instead, the literature reports a broad range of ideal selectivity, largely attributable to variations in dope formulation, solvent systems, fabrication parameters, and the molecular weight of the PSF employed [15]. The rise in CO_2_ permeability is primarily ascribed to the preferential sorption of CO_2_ molecules induced by the specific interactions between the oxygen containing functional groups on the GO nanosheets and CO_2_, as discussed in previous reports [33]. At GO loadings beyond this limit, a reduction in CO_2_ permeability is observed at 0.5 wt.%, arising from nanosheet agglomeration. Such aggregation at elevated nanofiller concentrations is reported in many studies to induce non-ideal morphology and transport pathways in MMMs, thereby diminishing their separation efficiency [34]. The agglomeration of GO nanosheets at a 0.5 wt.% loading induced interfacial void formation within the MMM structure, consequently compromising the integrity of the polymer filler interface and deteriorating the overall gas separation performance, consistent with earlier studies [25]. In contrast, CH_4_ permeability remains significantly lower throughout the series, decreasing slightly at 0.1 wt.% GO and exhibiting a minor rise at higher loadings. The diminished CH_4_ permeance can be attributed to the incorporation of GO nanosheets, which impose a more tortuous diffusion pathway and preferentially hinder the transport of larger molecules such as CH_4_. In contrast, the abundant oxygenated functional groups on GO promote specific interactions with polar CO_2_, thereby enhancing its permeance [35]. Owing to the absence of such interactions with non-polar CH_4_ exhibiting lower permeance, a trend consistent with prior reports [36].

The corresponding CO_2_/CH_4_ ideal selectivity, shown in Figure 6b, exhibits a pronounced improvement with increasing GO loading, attaining a peak value of 14.32 at 0.3 wt.% GO, followed by a reduction at 0.5 wt.% (8.04), which might be attributed to the agglomeration, or void formation on the surface, or may be the compactness of polymer chains, which reduces the fractional free volume of the membrane. The enhanced selectivity at moderate GO loadings attributed to strong interfacial interactions between PSF and GO, as supported by the FTIR analysis, which indicated hydrogen bonding and dipole–dipole interactions between the oxygenated groups of GO (–OH, –COOH, and –C=O) and the polar sulfone (–SO_2_–) and ether (C–O–C) functionalities of PSF. The improvement in CO_2_/CH_4_ selectivity can be ascribed to the enhanced CO_2_ permeability in PSF/GO MMMs, which exhibits strong sorption affinity toward CO_2_. The π–π stacking domains and oxygenated functionalities on GO promote preferential interactions with polarizable CO_2_, whereas non-polar CH_4_ experiences minimal affinity [37]. Although GO nanosheets are generally presumed to be randomly dispersed, partial alignment within the polymer matrix cannot be excluded. Such a microstructural arrangement may generate preferential diffusion channels that facilitate CO_2_ transport while imposing additional resistance to the migration of bulkier CH_4_ molecules, thereby increasing selectivity. Similar behaviour has been reported in the literature [38].

Furthermore, the inherently higher condensability and superior ideal solubility of CO_2_ (29.5 × 10^−3^ cm^3^(STP)/cm^3^·cmHg) relative to CH_4_ support its higher permeance. In contrast, CH_4_ exhibits a significantly lower solubility coefficient (18.46 × 10^−3^ cm^3^(STP)/cm^3^·cmHg), reflecting its reduced capacity to dissolve and diffuse through the polymer matrix, as reported by Baker (2004) [39]. Consequently, the pronounced increase in CO_2_ permeance, coupled with the suppressed CH_4_ transport, further intensified by the barrier effect introduced by GO nanosheets, enhances the CO_2_/CH_4_ selectivity of the MMM.

A comparison with literature data further validates the efficiency of GO as a functional filler in PSF-based MMMs. For instance, PSF/PEG grafted CNT MMMs exhibited a CO_2_ permeability of approximately 9 Barrer and CO_2_/CH_4_ selectivity of 10.2 at 5 wt.% filler loading [40], whereas PSF/NH_2_-MIL-125(Ti) MMMs achieved CO_2_ permeability in the range of 10–15 Barrer with a selectivity of 10–12 [41]. In contrast, the present PSF/GO-0.3 wt.% membrane demonstrates higher CO_2_ permeability (21.63 Barrer) and improved selectivity (14.32) at substantially lower filler content (0.3 wt.%), underscoring the superior interfacial compatibility and gas transport efficiency of GO within the PSF matrix. Similar enhancement trends have been reported for PSF/MCM-41 MMMs, where CO_2_ permeability increased from 5.8 to 12.5 Barrer as filler loading increased [42]. However, this required much higher filler content (~20 wt.%). These comparisons highlight the effectiveness of GO as a low concentration, high performance nanofiller that simultaneously enhances gas transport and selectivity by improving interfacial morphology and minimizing non-selective defects.

Overall, the obtained results confirm that incorporating an optimal amount of GO into PSF synergistically improves CO_2_ permeability and CO_2_/CH_4_ selectivity, surpassing the typical performance of pristine PSF and many reported MMMs in the literature. The formation of favourable interfacial interactions, improved CO_2_ sorption capability, and the creation of GO mediated diffusion channels all contribute to more efficient CO_2_ transport through the membrane matrix.

### 3.7. CO2-Induced Plasticization Effect

**Figure 7:**
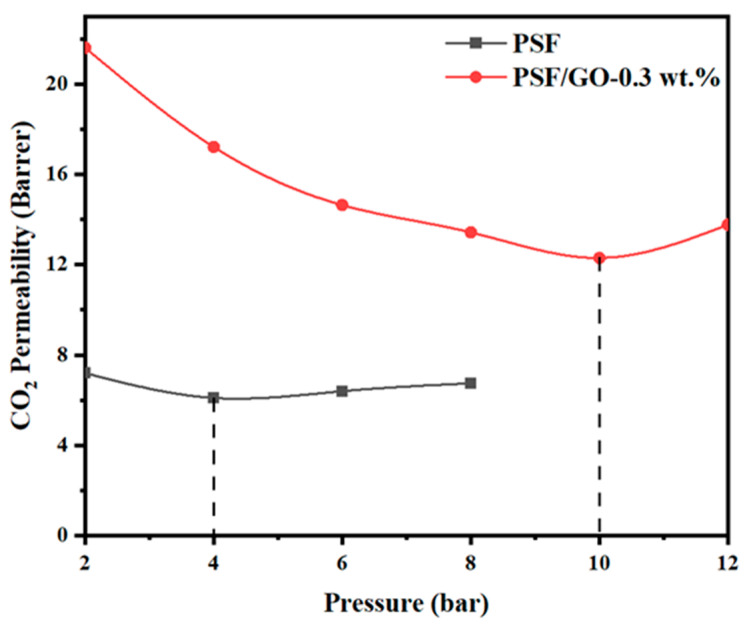
Effect of pressure on the CO_2_ permeability using PSF and PSF/GO-0.3 wt.%.

The variation in CO_2_ permeability with feed pressure for the pristine PSF and PSF/GO (0.3 wt.%) membranes is illustrated in Figure 7. For the pure PSF membrane, CO_2_ permeability initially increased with pressure up to approximately 4 bar, followed by a gradual decline at higher pressures (6 bar). This reduction is attributed to polymer chain densification under compression, which decreases the available free volume and limits gas transport. In contrast, the PSF/GO-0.3 wt.% membrane exhibited a more stable permeability profile over the pressure range of 2–10 bar, with only a slight decline observed. This behaviour indicates that GO incorporation effectively enhanced the structural rigidity of the polymer matrix and restricted chain mobility. The strong interfacial interactions between GO and PSF, primarily through hydrogen bonding between GO hydroxyl groups and PSF sulfone or ether groups, improve filler/matrix compatibility and suppress premature plasticization.

When the feed pressure was increased to 12 bar, a moderate increase in CO_2_ permeability was observed in the PSF/GO membrane, indicating the onset of CO_2_ induced plasticization. This phenomenon occurs when sorbed CO_2_ molecules penetrate the polymer matrix, increasing segmental mobility and expanding free volume, thereby enhancing gas diffusivity but potentially compromising selectivity. The delayed appearance of this effect in the PSF/GO membrane, compared with the pristine PSF, confirms that the addition of GO improves resistance to plasticization and enhances the overall stability of the membrane under high pressure CO_2_ exposure.

### 3.8. Comparison with Robson Upper Bound Relationship

**Figure 8:**
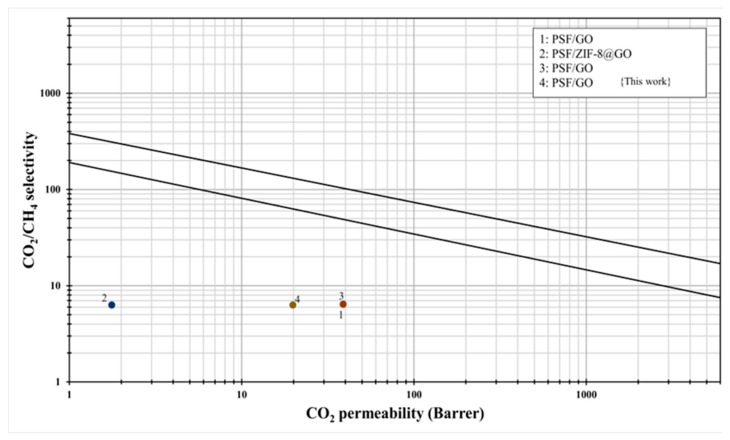
Robeson upper bound comparison with PSF/GO membranes.

The gas separation performance of the PSF/GO membranes was assessed in relation to the Robeson upper bound for CO_2_/CH_4_ separation (Figure 8). The pristine PSF membrane exhibited permeability/selectivity values lying below the 2008 Robeson upper-bound curve, consistent with the intrinsic trade-off between gas permeability and selectivity observed in conventional glassy polymers. Upon incorporation of GO nanosheets, a distinct improvement was observed. The PSF/GO (0.3 wt.%) membrane showed a concurrent improvement in permeability and CO_2_/CH_4_ selectivity, positioning its performance much closer to the Robeson 2008 upper bound line. Sainath et al. [33] recently developed PSF-based hollow fiber membranes (HFM) with improved long term performance stability by incorporating GO nanosheets. The HFM containing 0.25 wt.% GO demonstrated significant improvements in selectivity. Specifically, the membrane exhibited approximately 137% enhancement in ideal CO_2_/CH_4_ selectivity and a 201% increase in mixed gas CO_2_/CH_4_ selectivity compared to the unmodified membrane. Despite these advancements, the overall separation efficiency of such membranes still falls short of the Robeson upper bound. In a related study, Anastasiou et al. [15] developed ZIF-8 metal organic frameworks (MOFs) and ZIF-8/GO hybrid nanofillers and embedded them into a PSF matrix to fabricate MMMs. They conducted a comprehensive evaluation of the structural, morphological, and sorption properties of both the nanofillers and the resulting membranes, followed by an assessment of their CO_2_, N_2_, and CH_4_ permeation behavior. The inclusion of ZIF-8/GO hybrids significantly improved membrane performance, yielding an 87% increase in CO_2_ permeability and a 61% rise in CO_2_/CH_4_ selectivity compared to neat PSF. Nevertheless, these membranes also did not surpass the Robeson limit. Overall, the comparison suggests that integrating GO-based fillers into a PSF matrix effectively enhances both gas transport and selectivity, although the performance remains below the upper bound threshold.

## 4. Conclusions

PSF/GO MMMs were fabricated via solution blending and systematically characterized for their morphological, structural, and gas transport properties. The incorporation of GO nanosheets significantly influenced the membrane morphology and interfacial characteristics, as confirmed by SEM, EDX, FTIR, DSC, and TGA analyses. Uniform GO dispersion at moderate loading (0.3 wt.%) led to improved polymer filler compatibility through hydrogen bonding interactions amongst the GO oxygen containing groups and sulfone moieties of PSF, resulting in a dense, defect-free membrane structure. Gas permeation results demonstrated that the inclusion of GO enhanced both CO_2_ permeability and CO_2_/CH_4_ selectivity compared with the pristine PSF membrane. The improved selectivity was ascribed to the enhanced affinity of GO toward CO_2_ and the reduced development of non-selective voids within the matrix. Furthermore, the PSF/GO membrane exhibited superior resistance to CO_2_ induced plasticization, maintaining its structural integrity and stable permeability at higher feed pressures. The delayed onset of plasticization highlights the reinforcing role of GO, which restricts polymer chain mobility and suppresses swelling under high CO_2_ partial pressures. Overall, the results confirm that incorporating a small fraction of GO (0.3 wt.%) effectively enhances the separation performance and operational stability of PSF-based membranes. The developed PSF/GO MMMs offer a promising route toward efficient CO_2_/CH_4_ separation, with prospective applicability in natural gas purification and post-combustion carbon capture processes. Future work should focus on optimizing filler surface chemistry and evaluating long-term performance under realistic mixed gas and high pressure conditions.

## Data Availability

The original contributions presented in the study are included in the article. Further inquiries can be directed to the corresponding author.

## References

[1] Tabish A., Varghese A.M., Wahab M.A., Karanikolos G.N. (2020). Perovskites in the Energy Grid and CO_2_ Conversion: Current Context and Future Directions. Catalysts.

[2] Varghese A.M., Karanikolos G.N. (2020). CO_2_ capture adsorbents functionalized by amine–bearing polymers: A review. Int. J. Greenh. Gas Control.

[3] Yu S., Li C., Zhao S., Chai M., Hou J., Lin R. (2024). Recent advances in the interfacial engineering of MOF-based mixed matrix membranes for gas separation. Nanoscale.

[4] Imran M., Lau K.K., Ahmad F., Mohd Laziz A. (2025). A comprehensive review of Computational Fluid Dynamics (CFD) modelling of membrane gas separation process. Results Eng..

[5] Muthukumaraswamy Rangaraj V., Wahab M.A., Reddy K.S.K., Kakosimos G., Abdalla O., Favvas E.P., Reinalda D., Geuzebroek F., Abdala A., Karanikolos G.N. (2020). Metal organic framework—Based mixed matrix membranes for carbon dioxide separation: Recent advances and future directions. Front. Chem..

[6] Favvas E.P., Heliopoulos N.S., Karousos D.S., Devlin E., Papageorgiou S.K., Petridis D., Karanikolos G.N. (2019). Mixed matrix polymeric and carbon hollow fiber membranes with magnetic iron-based nanoparticles and their application in gas mixture separation. Mater. Chem. Phys..

[7] Pazani F., Maleh M.S., Shariatifar M., Jalaly M., Sadrzadeh M., Rezakazemi M. (2022). Engineered graphene-based mixed matrix membranes to boost CO_2_ separation performance: Latest developments and future prospects. Renew. Sustain. Energy Rev..

[8] Kadirkhan F., Goh P.S., Ismail A.F., Wan Mustapa W.N.F., Halim M.H.M., Soh W.K., Yeo S.Y. (2022). Recent advances of polymeric membranes in tackling plasticization and aging for practical industrial CO_2_/CH_4_ applications—A review. Membranes.

[9] Mahenthiran A.V., Jawad Z.A., Chin B.L.F. (2023). Development of blend PEG-PES/NMP-DMF mixed matrix membrane for CO_2_/N_2_ separation. Environ. Sci. Pollut. Res..

[10] Zhang Y., Sunarso J., Liu S., Wang R. (2013). Current status and development of membranes for CO_2_/CH_4_ separation: A review. Int. J. Greenh. Gas Control.

[11] Han Y., Ho W.W. (2021). Polymeric membranes for CO_2_ separation and capture. J. Membr. Sci..

[12] Goh P., Ismail A., Sanip S., Ng B., Aziz M. (2011). Recent advances of inorganic fillers in mixed matrix membrane for gas separation. Sep. Purif. Technol..

[13] Goh S.H., Lau H.S., Yong W.F. (2022). Metal–organic frameworks (MOFs)-based mixed matrix membranes (MMMs) for gas separation: A review on advanced materials in harsh environmental applications. Small.

[14] Alkhattabi L., Harun N.Y., Zeeshan M.H., Waqas S., Hanbazazah A. (2025). Gradient Cross-Linking Graphene Oxide–Integrated Nanofiltration Polyvinylpyrrolidone Membrane for Polycyclic Aromatic Hydrocarbons Removal. Int. J. Polym. Sci..

[15] Anastasiou S., Bhoria N., Pokhrel J., Reddy K.S.K., Srinivasakannan C., Wang K., Karanikolos G.N. (2018). Metal-organic framework/graphene oxide composite fillers in mixed-matrix membranes for CO_2_ separation. Mater. Chem. Phys..

[16] Douna I., Farrukh S., Hussain A., Salahuddin Z., Noor T., Pervaiz E., Younas M., Fan X.F. (2022). Experimental investigation of polysulfone modified cellulose acetate membrane for CO_2_/H_2_ gas separation. Korean J. Chem. Eng..

[17] Elyasi Kojabad M., Momeni M., Babaluo A.A., Vaezi M.J. (2020). PEBA/PSf multilayer composite membranes for CO_2_ separation: Influence of dip coating parameters. Chem. Eng. Technol..

[18] Abdulabbas A.A., Mohammed T.J., Al-Hattab T.A. (2024). Parameters estimation of fabricated polysulfone membrane for CO_2_/CH_4_ separation. Results Eng..

[19] Natarajan P., Sasikumar B., Elakkiya S., Arthanareeswaran G., Ismail A., Youravong W., Yuliwati E. (2021). Pillared cloisite 15A as an enhancement filler in polysulfone mixed matrix membranes for CO_2_/N_2_ and O_2_/N_2_ gas separation. J. Nat. Gas Sci. Eng..

[20] Singh S., Varghese A.M., Reinalda D., Karanikolos G.N. (2021). Graphene—Based membranes for carbon dioxide separation. J. CO_2_ Util..

[21] Yoo B.M., Shin J.E., Lee H.D., Park H.B. (2017). Graphene and graphene oxide membranes for gas separation applications. Curr. Opin. Chem. Eng..

[22] Pokhrel J., Bhoria N., Wu C., Reddy K.S.K., Margetis H., Anastasiou S., George G., Mittal V., Romanos G., Karonis D. (2018). Cu-and Zr-based metal organic frameworks and their composites with graphene oxide for capture of acid gases at ambient temperature. J. Solid State Chem..

[23] Li W., Xu P., Wang Z., He Y., Qin H., Zeng Y., Li Y., Zhang Z., Gao J. (2023). MOFs meet membrane: Application in water treatment and separation. Mater. Chem. Front..

[24] Wong K.C., Goh P.S., Ismail A.F. (2017). Highly permeable and selective graphene oxide-enabled thin film nanocomposite for carbon dioxide separation. Int. J. Greenh. Gas Control.

[25] Berean K.J., Ou J.Z., Nour M., Field M.R., Alsaif M.M., Wang Y., Ramanathan R., Bansal V., Kentish S., Doherty C.M. (2015). Enhanced gas permeation through graphene nanocomposites. J. Phys. Chem. C.

[26] Lin Z., Yuan Z., Dai Z., Shao L., Eisen M.S., He X. (2023). A review from material functionalization to process feasibility on advanced mixed matrix membranes for gas separations. Chem. Eng. J..

[27] Ma Y., Zhang F., Lively R.P. (2020). Manufacturing nanoporous materials for energy-efficient separations: Application and challenges. Sustainable Nanoscale Engineering.

[28] Zeeshan M.H., Ruman U.E., Shafiq M., Waqas S., Sabir A. (2024). Intercalation of GO-Ag nanoparticles in cellulose acetate nanofiltration mixed matrix membrane for efficient removal of chromium and cobalt ions from wastewater. J. Environ. Chem. Eng..

[29] Zeeshan M.H., Yeong Y.F., Chew T.L. (2025). A comprehensive study on the effect of air gap distances on morphology and gas separation performance of cellulose triacetate/polysulfone dual-layer hollow fiber membrane. J. Chem. Technol. Biotechnol..

[30] Mahmoud K.A., Mansoor B., Mansour A., Khraisheh M. (2015). Functional graphene nanosheets: The next generation membranes for water desalination. Desalination.

[31] Zahri K., Wong K.C., Goh P.S., Ismail A.F. (2016). Graphene oxide/polysulfone hollow fiber mixed matrix membranes for gas separation. RSC Adv..

[32] Wan C., Chen B. (2012). Reinforcement and interphase of polymer/graphene oxide nanocomposites. J. Mater. Chem..

[33] Sainath K., Modi A., Bellare J. (2021). CO_2_/CH_4_ mixed gas separation using graphene oxide nanosheets embedded hollow fiber membranes: Evaluating effect of filler concentration on performance. Chem. Eng. J. Adv..

[34] Aroon M., Ismail A., Matsuura T., Montazer-Rahmati M. (2010). Performance studies of mixed matrix membranes for gas separation: A review. Sep. Purif. Technol..

[35] Shen G., Zhao J., Guan K., Shen J., Jin W. (2017). Highly efficient recovery of propane by mixed-matrix membrane via embedding functionalized graphene oxide nanosheets into polydimethylsiloxane. AlChE J..

[36] Hu C.-C., Chang C.-S., Ruaan R.-C., Lai J.-Y. (2003). Effect of free volume and sorption on membrane gas transport. J. Membr. Sci..

[37] Cruz-Silva R., Endo M., Terrones M. (2016). Graphene oxide films, fibers, and membranes. Nanotechnol. Rev..

[38] Chae I.S., Lee J.H., Hong J., Kang Y.S., Kang S.W. (2014). The platform effect of graphene oxide on CO_2_ transport on copper nanocomposites in ionic liquids. Chem. Eng. J..

[39] Baker R.W. (2004). Membrane technology and applications.

[40] Singh S., Varghese A.M., Reddy K.S.K., Romanos G.E., Karanikolos G.N. (2021). Polysulfone mixed-matrix membranes comprising poly (ethylene glycol)-grafted carbon nanotubes: Mechanical properties and CO_2_ separation performance. Ind. Eng. Chem. Res..

[41] Alsaady M., Waqas S., Zeeshan M.H., Almarshoud M.A., Maqsood K., Abdulrahman A., Yan Y. (2025). Efficient CO_2_/CH_4_ Separation Using Polysulfone/NH_2_-MIL-125(Ti) Mixed Matrix Membranes. ACS Omega.

[42] Miricioiu M.G., Iacob C., Nechifor G., Niculescu V.-C. (2019). High selective mixed membranes based on mesoporous MCM-41 and MCM-41-NH_2_ particles in a polysulfone matrix. Front. Chem..

